# *Brassica napus PHR1* Gene Encoding a MYB-Like Protein Functions in Response to Phosphate Starvation

**DOI:** 10.1371/journal.pone.0044005

**Published:** 2012-08-29

**Authors:** Feng Ren, Qian-Qian Guo, Li-Li Chang, Liang Chen, Cai-Zhi Zhao, Hui Zhong, Xue-Bao Li

**Affiliations:** Hubei Key Laboratory of Genetic Regulation and Integrative Biology, College of Life Sciences, Central China Normal University, Wuhan, China; Centro de Investigación y de Estudios Avanzados del IPN, Mexico

## Abstract

Phosphorus (P) is one of the essential nutrient elements for plant development. In this work, *BnPHR1* encoding a MYB transcription activator was isolated from *Brassica napus*. The characterization of nuclear localization and transcription activation ability suggest BnPHR1 is a transcriptional activator. The tissue expression and histochemical assay showed that *BnPHR1* was predominantly expressed in roots and modulated by exogenous Pi in transcriptional level in roots under Pi deficiency conditions. Furthermore, overexpression of *BnPHR1* in both Arabidopsis and *B. napus* remarkably enhanced the expression of the Pi-starvation-induced genes including *ATPT2* and *BnPT2* encoding the high-affinity Pi transporter. Additionally, BnPHR1 can *in vivo* bind the promoter sequence of *ATPT2* and *BnPT2* in both Arabidopsis and *B. napus*. Possibly, due to the activation of ATPT2 and BnPT2, or even more high-affinity Pi transporters, the excessive Pi was accumulated in transgenic plants, resulting in the crucially Pi toxicity to cells and subsequently retarding plant growth. Given the data together, BnPHR1, as crucial regulator, is regulated by exogenous Pi and directly activates those genes, which promote the uptake and homeostasis of Pi for plant growth.

## Introduction

Phosphorus (P) is one of essential nutrient elements for plant growth and development. Although abundant P is present in a lot of soils, very little of it is present in phosphate (Pi) forms that are available for plants [1]. To adapt the low availability of Pi, plants have evolved complicated mechanisms [2]. Morphologically, the growth and architecture of root is modified, including increased root-shoot ratio and proliferation of long root hairs to enhance total surface area available for soil exploration [3], [4]. In addition, some plants can improve their soil scavenging capacity by forming clusters of lateral roots or by establishing symbiotic associations with mycorrhizal fungi [2]. In soil in which Pi is distributed in patches, lateral roots proliferate in the high Pi areas and are inhibited in the low Pi areas [5]. The roots of P-deficient plants could exude organic acids into soil solution to increase the availability of phosphate bound to soil particles [6]. Plants also use endogenous P by increasing the activity of enzymes that replace P in metabolites and structural compounds due to Pi deficiency [2], [7].

The molecular regulation of plants responding to Pi deficiency remains unclear so far, although some genes, encoding Pi transporters, phosphatases, RNases, transcription factor, and others of unknown function that help plants adapt to low Pi, have been characterized [8]. To increase Pi uptake capacity, the transcripts and activity of high affinity phosphate transporters are induced to optimize uptake and remobilization of phosphate in P-deficient plants [2]. The Pi transporters may play a crucial role in uptake and use of Pi, as the transport of Pi across membranes is an important step in this process. The high-affinity Pi transporters are membrane-associated proteins which can translocate Pi from an external media containing µM concentrations to the cytoplasm [7]. Some Pi transporters have been identified in plants, such as tomato and Arabidopsis [9]–[11]. The RNases are presumed to release Pi from RNA molecules in the extracellular matrix including those derived from other organisms in the rhizosphere or present within cells. Under Pi starvation conditions, the genes encoding RNases, are induced in plants [12], [13]. On the other hand, these Pi-starvation responsive genes are transcriptionally regulated by transcription factors. AtPHR1, one of MYB-CC family transcription factors, is a master transcriptional activator of the Pi-starvation response in Arabidopsis [14]. AtPHR1 regulates a number of Pi starvation-induced genes through binding a P1BS (PHR1 specific binding sequence) *cis*-element (GNATATNC), which is present in the promoter regions of Pi starvation-induced genes [14]. The *phr1* mutant displays a reduced concentration of Pi under both Pi-sufficient and Pi-deficient conditions and minimal induction of anthocyanin accumulation in response to Pi deprivation [14], [15]. On the contrary, *AtPHR1* overexpression leads to an increased concentration of Pi in the shoots and the elevated expression of a range of Pi starvation-induced genes that encode the Pi transporter, phosphatase and RNase [15]. *PHR1-LIKE1* (*PHL1*), another member of MYB-CC family, is redundant with *PHR1* acting as central integrators of Pi starvation responses [16]. The homologues of *AtPHR1* have been found in rice and common bean. Rice OsPHR1 and OsPHR2 were identified to be involved in regulating several Pi-starvation induced genes. However, only overexpression of *OsPHR2* results in increased Pi accumulation in shoots of transgenic rice [17]. In common bean, PvPHR1 is a positive regulator of genes implicated in Pi transport, remobilization and homeostasis [18]. Taken together, the data suggest that PHR1 is a master transcriptional activator in controlling Pi uptake and allocation in plants.

However, both the transcript level of *PHR1* gene and nuclear localization of PHR1 protein are not altered significantly by Pi status in *Arabidopsis* and rice [14], [17]. Therefore, the post-transcriptional modification was hypothesized to be involved in PHR1 activity. Supporting this notion, AtPHR1 was revealed to be sumoylated by AtSIZ1, a SUMO E3 ligase [19]. The sumoylation involved in various cellular processes can stabilize the target proteins and alter their sub-cellular localization [20]. The *siz1* mutant exhibits hypersensitive responses to Pi starvation, including changes in root architecture with reduced primary root growth and the massive formation of lateral roots and root hairs, an increased ratio of root-to-shoot mass and greater anthocyanin accumulation [19]. The intracellular Pi concentration is higher in shoots of *siz1* mutant than that of wild-type plants under Pi-sufficient conditions, possibly because of enhanced expression of PHT1;4 Pi transporter [19]. AtSIZ1 may act as a negative as well as a positive regulator. As one of AtPHR1 targets, miRNA399 is specifically induced by Pi starvation and reciprocally regulates *PHO2* gene encoding an ubiquitin conjugating E2 enzyme at the transcriptional level [19], [21]. The loss-of-function mutation of *PHO2* leads to excessive accumulation of Pi in shoot tissues [21], [22].

In addition to PHR1, members of bHLH, WRKY, Zinc finger and R2R3 MYB families of transcription factors are involved in the control of Pi starvation responses, although their exact positions in the signaling pathway have not been established. AtMYB62, another MYB transcription factor, as a negative regulator of other Pi starvation-inducible genes, is involved in the Pi deprivation response of Arabidopsis [23]. OsPTF1, a bHLH transcription factor, was identified in rice roots and found to be responsive to Pi deficiency [24]. AtZAT6, a cysteine-2/histidine-2 (C2H2) zinc finger transcription factor, appears to be a repressor of primary root growth, regulating Pi homeostasis through the control of root architecture [25]. AtWRKY75, a member of the WRKY transcription factor family, is induced in Pi deficiency [26]. AtWRKY6, another member of the WRKY transcription factor family, negatively regulates *PHO1* expression by binding to two W-box consensus motifs within the *PHO1* promoter, and the repression of *PHO1* expression by WRKY6 is released under low Pi conditions [27].

In this study, *BnPHR1* gene was identified in *B. napus*. To investigate the role of *BnPHR1* in Pi-starvation response, we developed transgenic lines with overexpression of *BnPHR1* in Arabidopsis and *B. napus* for Pi-signaling and Pi-uptake analysis. Our results revealed that BnPHR1 is involved in the Pi-signaling pathway, and the overexpression of BnPHR1 results in Pi accumulation in shoots of transgenic plants. As a crucial regulator of Pi starvation response in *B. napus*, BnPHR1 directly activated *PT2* encoding the high-affinity Pi transporter which promote the uptake of Pi for plant growth.

## Materials and Methods

### Plant Growth Conditions

*Brassica napus* (cv. Westar) and Arabidopsis seeds were surface sterilized and germinated on half-strength Murashige and Skoog (MS) medium (pH 5.8) containing 0.8% agar at 22–25°C. After 10 days, germinated *B. napus* and Arabidopsis seedlings were transferred into soil in the greenhouse. Plants were grown under a long-day light cycle (16 h light/8 h dark) and the humidity was maintained at 65%.

### Gene Cloning, Vector Construction and Plant Transformation

From Pi starvation induced genes in *B. napus*, one clone was identified as homologous gene of *AtPHR1* and consequently designated as *BnPHR1*, which may be the major regulator of Pi-starvation response in *B. napus*. With the gene-specific primers (BnPHR1F/R, Table 1), the open reading frame of *BnPHR1* was amplified and then cloned into pBluescript SK vector for sequencing verification.

**Table 1:** Gene-specific primers or oligonucleotides used in this paper.

Name	Sequences
*BnPHR1F*	gctctagaatggaggctcgtccggttc
*BnPHR1R*	gcggatcctcagttatcggttttggg
*BnPHR1RT1*	gagggatgggtatcaccgag
*BnPHR1RT2*	tcagttatcggttttggggcg
*BnPHR1p1*	gcaagcttgaggcagagataatgaaaacg
*BnPHR1p2*	cggatccgacctaaatgaatttaagttggc
*LBa1*	tggttcacgtagtgggccatcg
*LBb1*	gcgtggaccgcttgctgcaact
*LP629*	gagagacctcacacgcacttc
*RP629*	ctttctggcgaacctgtagtg
*phr1-f*	gaagtacagaagcaactccatgagcagctc
*phr1-r*	tcaattatcgattttgggacg
*BnACT2F*	gtgttgttggtaggccaagacatca
*BnACT2R*	cttgatgtctcttacaatttcccgc
*AtACT2F*	gaaatcacagcacttgcacc
*AtACT2R*	aagcctttgatcttgagagc
*ATPT2p1*	acgagtgtggactaatattgagttgac
*ATPT2p2*	gttgttaacgacaacttctcatgttcc
BnPT2p1	aattggaggggaatataccgaaacattgttgtatattcatgtaag
*BnPT2p2*	ccggcttacatgaatatacaacaatgtttcggtatattcccctcc
*BnPT2RT1*	gtaccggcggagatcttcccagc
*BnPT2RT2*	ctacacaatggggaccgttc
*IPS1RT1*	tctatcctttggcaagcttcggttc
*IPS1RT2*	catgcactggtctgactattctccaaac
*ATPT1RT1*	ccattgttggagcctttgggttc
*ATPT1RT2*	gaaaacaaaaccaaacatcgcactcc
*ATPT2RT1*	attaggagcaatggttggtg
*ATPT2RT2*	ctaaactattgggaccgttc
*ATPT2RT3*	gtaccaagaaagtgaagag
*ACP5RT1*	ggcttggagaggagatattaacccagtg
*ACP5RT2*	ggaagcctacctagcctgcaataggac
*PHO1RT1*	gagattcagggtcagtcctgttcagtctc
*PHO1RT2*	gatgcaaaatccgaaatgcttcctc
*RNS1RT1*	ctaaccaaagccgggattaatccg
*RNS1RT2*	gatcgatgccggttcaagagactg

The coding sequence of *BnPHR1* was cloned into pBI121 vector, replacing *GUS* gene. The pBI121-BnPHR1 construct was introduced into Arabidopsis by floral dip method. The transformed seeds were selected on MS medium containing 50 mg/L kanamycin. Homozygous lines of T3 and T4 generations were used for low Pi treatment and phenotype analysis.

The pBI121-BnPHR1 construct was also used for *B. napus* transformation. An *Agrobacterium tumefaciens*–mediated *B. napus* transformation system was modified from the above Arabidopsis floral dip method. The transgenic seeds were selected on MS medium containing 400 mg/L kanamycin. T2 generation transgenic plants were used for analyses of phenotype, genes expression and Pi content.

**Figure 1:**
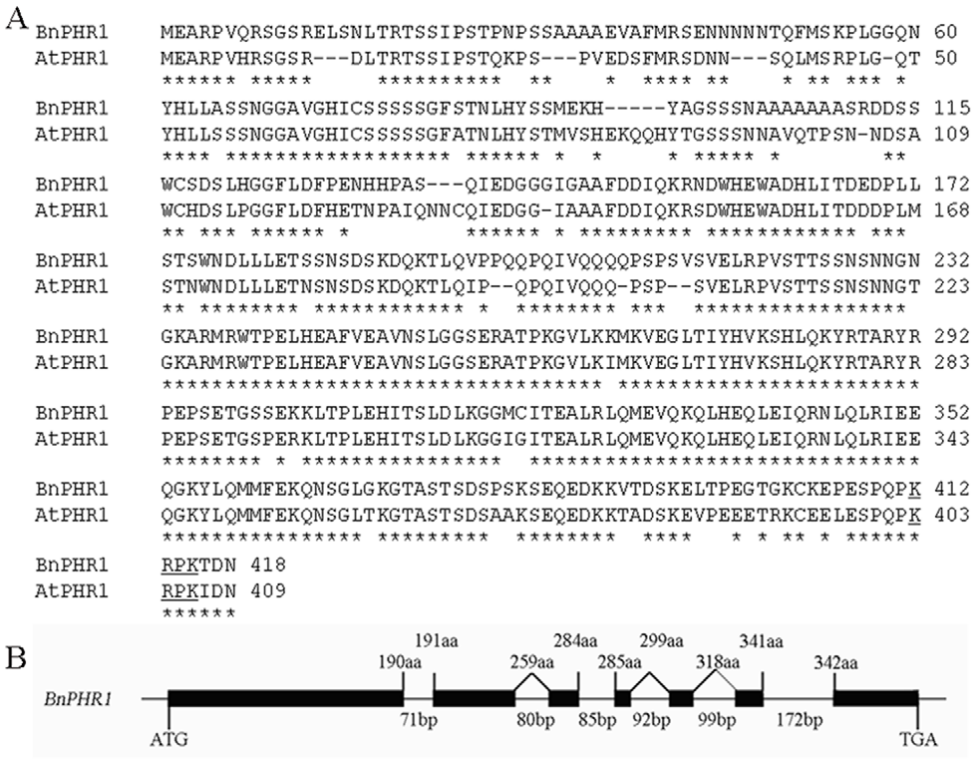
The characterization of *BnPHR1* gene and its deduced protein. (**A**) Comparison of amino acid sequences between BnPHR1 and AtPHR1. Amino acid sequences are aligned by ClusterW. The two conserved regions (MYB domain and coiled-coil domain) are highlighted in reverse contrast and gray, respectively. A putative nuclear localization signal is shown by underline. The asterisks show the positions of the conserved amino acids residues between BnPHR1 and AtPHR1. (**B**) Structure of *BnPHR1* gene. Exons are denoted by black boxes. Intron, 5′-untranslated region and 3′-untranslated region are denoted by lines. The length of the intron in base pairs is indicated. The number at the boundaries of each exon indicates the codon at which the intron is located. The translation initiation and termination codons are shown.

### Identification of Arabidopsis *phr1* Mutant and Complementation of *BnPHR1*

The *phr1* mutant (SALK_067629) was obtained from the SALK lines collection. The homozygous *phr1* mutant, which was selected by PCR-based genomic identification with four primers (LBa1, LBb1, LP629 and RP629, Table 1), was confirmed by RT-PCR. The coding sequence of *BnPHR1* was cloned into the modified pCAMBIA1301 vector with hygromycin resistance and introduced into *phr1* mutant. The transformed seeds were selected on MS medium containing 250 mg/L hygromycin.

**Figure 2:**
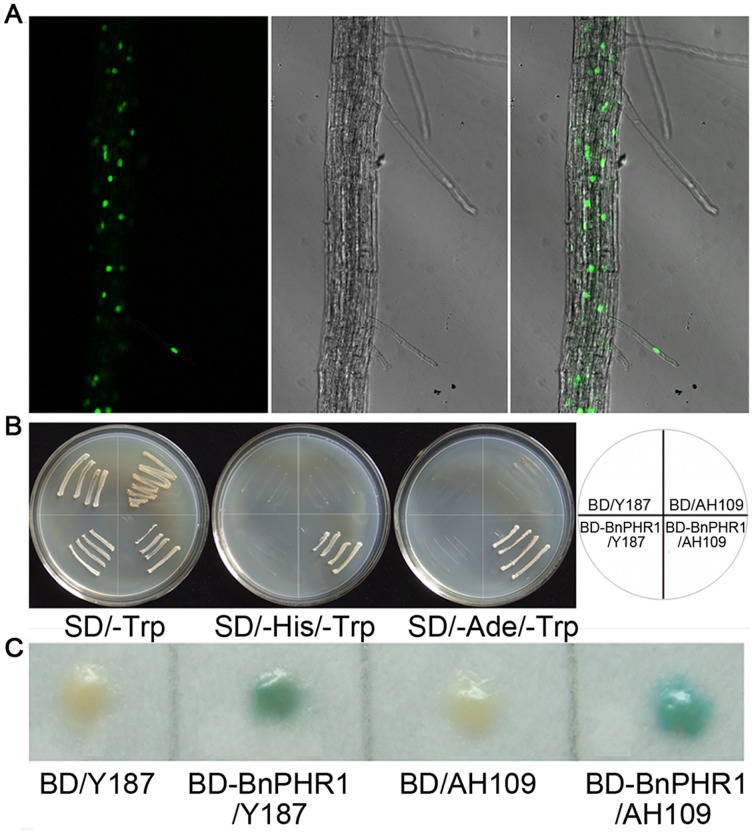
Nuclear localization and transcriptional-activation of BnPHR1. (**A**) Microscopic images of root cells of *35S:BnPHR1-GFP* transgenic Arabidopsis. Confocal images were taken under the GFP channel (left), and with transmitted light (midst), and the images of left and midst were merged (right). (**B** and **C**) Transcriptional activation of BnPHR1. The growth of yeast strain AH109 and Y187 with BD and BD-BnPHR1 constructs under SD/−Trp, SD/−His/−Trp and SD/−Ade/−Trp nutrition-deficient medium (**B**). The transcription activity of BnPHR1 was measured by α-galactosidase activity assay (**C**).

### Isolation of BnPHR1 Promoter and Histochemical Assay of GUS Activity

*BnPHR1* promoter was isolated from *B. napus* using Genome Walking Kit (TaKaRa, Dalian, China). A 1,265 bp 5′-flanking fragment of *BnPHR1* gene was amplified by PCR, using a pair of primers (BnPHR1p1/2, Table 1). *BnPHR1* promoter fragment was subcloned into pBI101 vector to generate the chimeric *BnPHR1P:GUS* construct. The *BnPHR1P:GUS* transgenic Arabidopsis was obtained by the floral dip method described as above.

**Figure 3:**
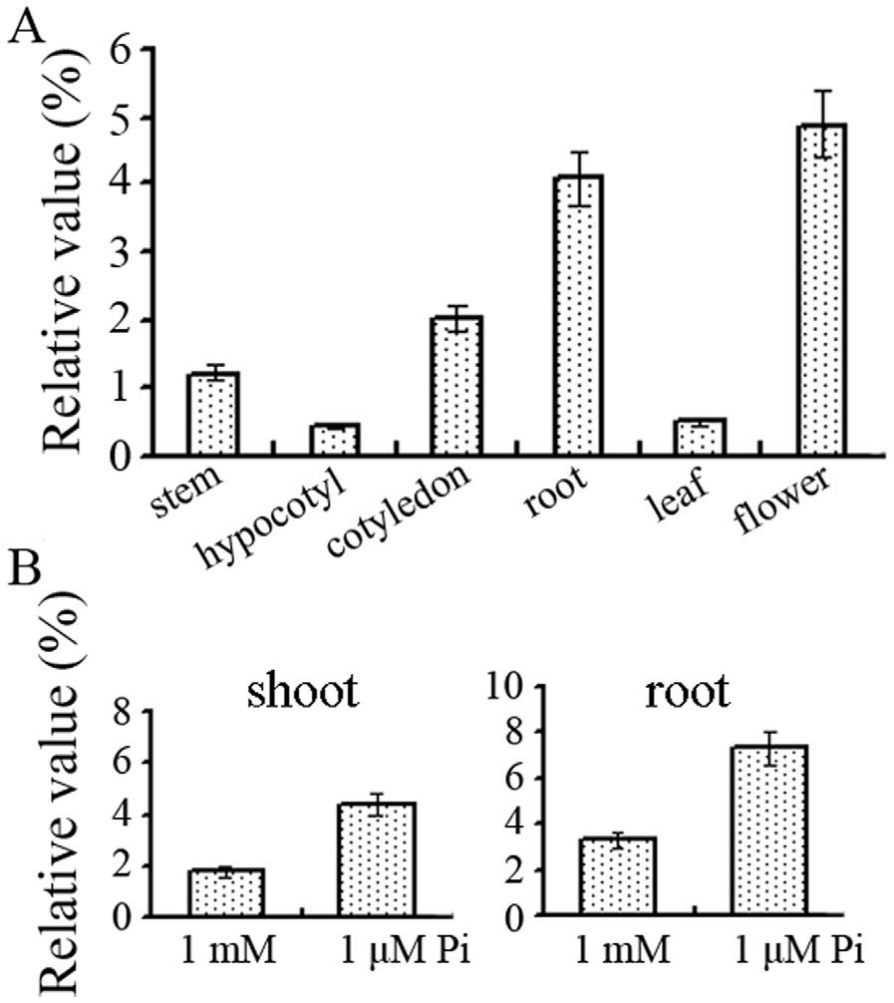
qRT-PCR analysis of *BnPHR1* expression in tissues of *B. napus*. (**A**) The wild-type *B. napus* plants were cultured in 1 mM Pi, and then the total RNAs isolated from stems, hypocotyls, cotyledons, roots, leaves and flowers were used in quantitative RT–PCR analysis. Error bars indicate standard deviation (n = 3). (**B**) The five-day-old *B. napus* seedling transferred to 1 µM Pi for 72 h and then total RNAs isolated from shoots and roots were used in quantitative RT–PCR analysis. Error bars indicate standard deviation (n = 3).

**Figure 4:**
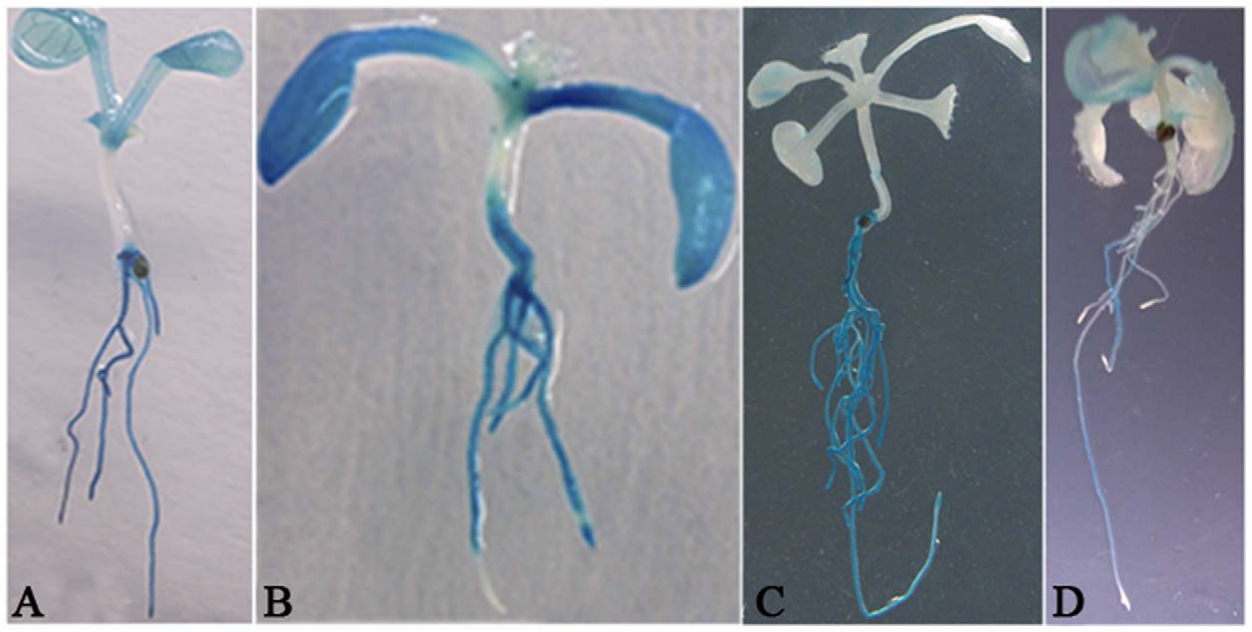
Histochemical assay of *BnPHR1* promoter activities in transgenic Arabidopsis. (**A**) The five-day-old seedling germinated on 1 mM Pi. (**B**) The five-day-old seedling transferred to 1 µM Pi for 72 h. (**C**) The eight-day-old seedling transferred to 1 mM Pi for 72 h. (**D**) The eight-day-old seedlings transferred to 1 µM Pi for 72 h.

Histochemical assays of GUS activity in transgenic Arabidopsis were conducted according to a modified protocol [28]. The five-day-old and eight-day-old seedlings of *BnPHR1P:GUS* transgenic Arabidopsis were transferred on MS with 1 mM and 1 µM Pi to vertically growth for 72 h. The samples were incubated at 37°C 2–4 h in a GUS reaction buffer including 5-bromo-4-chloro-3-indolyl-b-D-glucuronic acid (X-Gluc). Chlorophyll was cleared from plant tissues by immersing them in 70% ethanol. GUS staining patterns were confirmed by observing at least five different transgenic lines. The representative stained seedlings or tissues were imaged using a Leica MZ16f stereomicroscope (Leica, Germany).

**Figure 5:**
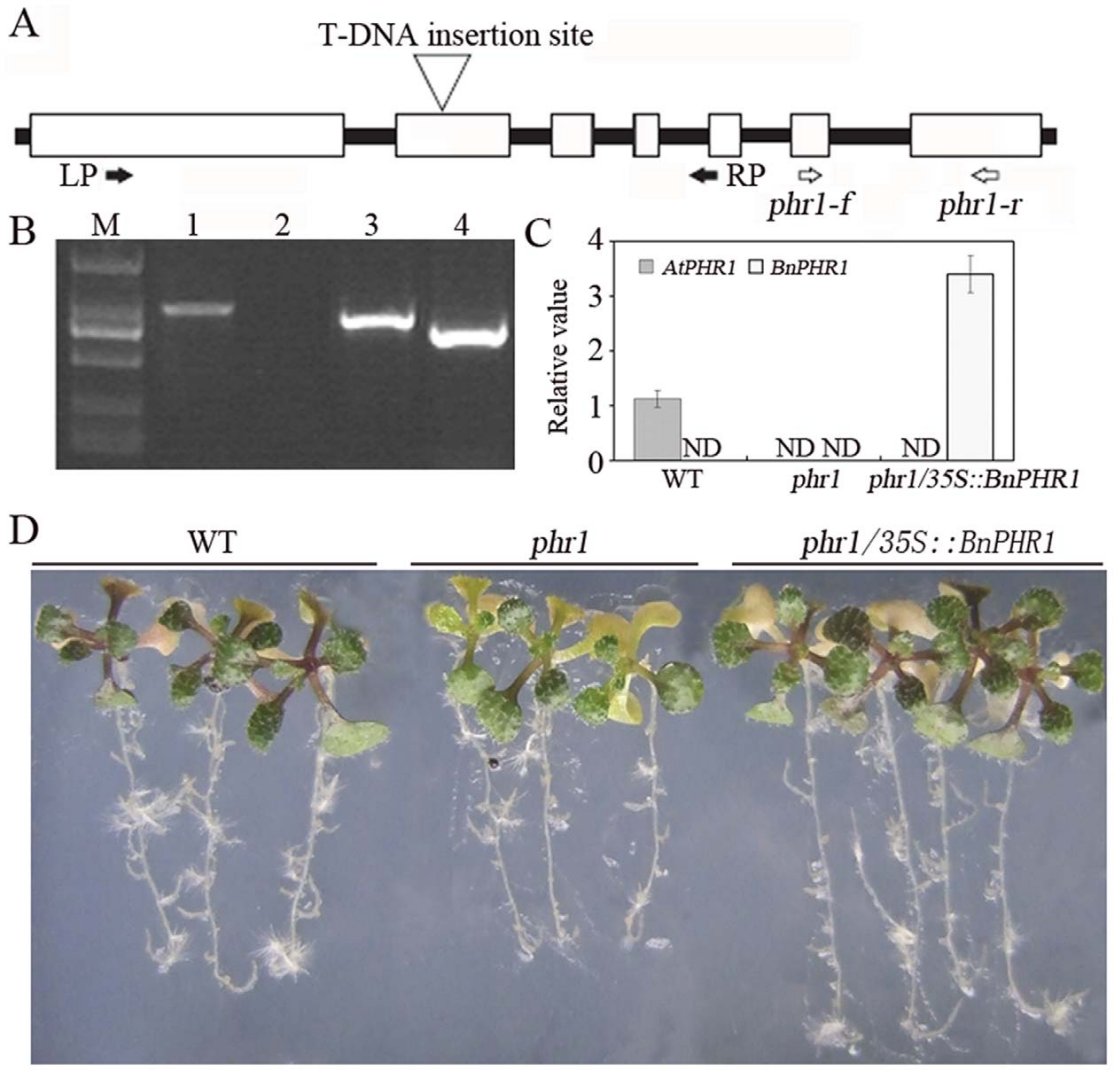
*BnPHR1* rescues the phenotypic defects of *phr1*. (**A**) The location of the T-DNA insertion site of the *phr1* mutant SALK_067629 is indicated with a triangle, and the locations of primers used for screening (LP(LP629) and RP(RP629), Table 1), and for RT–PCR (phr1-f, phr1-r, Table 1) are indicated with black and white arrows, respectively. (**B**) PCR analysis of the *phr1* mutant to verify homozygozity line. M, DNA marker; 1, PCR products with the primers LP629 and RP629 using wild type genomic DNA as templates; 2, no PCR products with the primers LP629 and RP629 using homozygous *phr1* genomic DNA as templates; 3, PCR products with the primers RP629 and LBa1 using homozygous *phr1* genomic DNA as templates; 4, PCR products with the primers RP629 and LBb1 using homozygous *phr1* genomic DNA as templates. (**C**) Relative expression of *AtPHR1* and *BnPHR1* in wild type (WT), *phr1* and *phr1/35S:BnPHR1* lines by real-time qRT–PCR using gene-specific primers (phr1-f/phr1-r and BnPHR1RT1/2, Table 1). Error bars indicate standard deviation (n = 3). ND indicates not detectable. (**D**) The phenotype of wild type (WT), *phr1* and *phr1/35S:BnPHR1* lines in MS with 1 µM Pi.

### Subcellular Localization and Transcriptional Activity Analysis

The coding sequence of *BnPHR1* was cloned into a pBI-eGFP vector to obtain *35S:BnPHR1-GFP* construct, and introduced in Arabidopsis by the floral dip method described as above. The transgenic seedlings were transferred to 1 µM Pi for 72 h. Subsequently, fluorescence microscopy was performed on a SP5 Meta confocal laser microscope (Leica, Germany). The roots of the transgenic seedlings were examined with a filter set for GFP fluorescence (488 nm for excitation and 506∼538 nm for emission). SP5 software (Leica, Germany) was employed to record and process the digital images taken.

**Figure 6:**
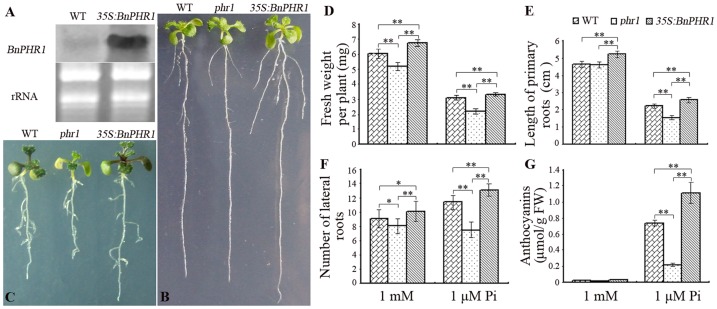
The promoted growth of *BnPHR1* overexpression Arabidopsis seedlings. (**A**) Northern blotting analysis of *BnPHR1* transcripts in *35S:BnPHR1* transgenic Arabidopsis. (**B**) The ten-day-old wild type (WT), *phr1* and *35S:BnPHR1* transgenic Arabidopsis seedlings in 1 mM Pi. (**C**) The ten-day-old wild type (WT), *phr1* and *35S:BnPHR1* transgenic Arabidopsis seedlings in 1 µM Pi. (**D** to **G**) The fresh weight (**D**), length of primary root (**E**), number of lateral roots (**F**) and anthocyanins content (**G**) of ten-day-old wild type (WT), *phr1* and *35S:BnPHR1* transgenic Arabidopsis seedlings in 1 mM Pi and 1 µM Pi. Error bars indicate standard deviation (*0.01*<p<*0.05, **p<0.01, n = 20).

The coding sequence of *BnPHR1* was cloned into pGBKT7 (Biosciences Clontech, Palo Alto, CA, USA) which containing GAL4 DNA binding domain (BD). The BD-BnPHR1 construct was transformed into the yeast strain AH109 and Y187, and three reporter genes *HIS*, *ADE* and *lacZ* were tested by streaking the yeast AH109 and Y187 transformants on SD/−Trp/−His and SD/−Trp/−Ade medium (SD minimal medium lacking Trp and His or lacking Trp and Ade) (Clontech Inc., Palo Alto, CA, USA) and by the flash-freezing filter assay of yeast AH109 and Y187 transformants, respectively [29].

**Figure 7:**
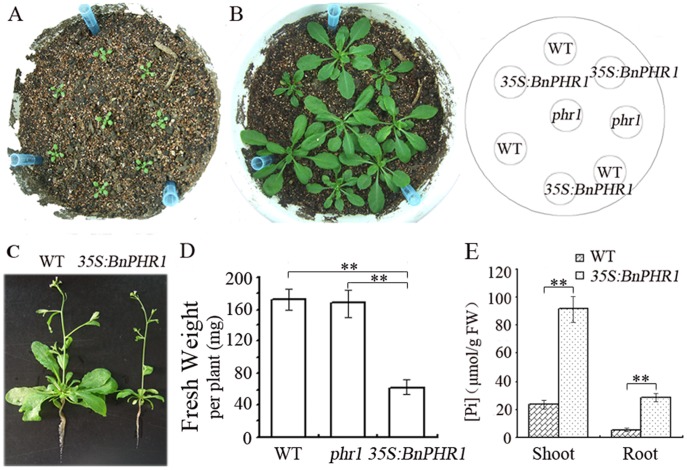
The retarded growth of *BnPHR1* overexpressing Arabidopsis in soil. (**A**) The one-week-old wild type (WT), *phr1* mutant and *35S:BnPHR1* transgenic Arabidopsis in soil. (**B**) The two-week-old wild type (WT), *phr1* mutant and *35S:BnPHR1* transgenic Arabidopsis in soil. (**C**) The bolting wild type (WT) and *35S:BnPHR1* transgenic Arabidopsis in soil. (**D**) The fresh weight of two-week-old wild type (WT), *phr1* and *35S:BnPHR1* transgenic Arabidopsis seedlings in soil. (**E**) The Pi content in shoot and root of wild type (WT) and *35S:BnPHR1* transgenic Arabidopsis. Error bars indicate standard deviation (*0.01*<p<*0.05, **p<0.01, n = 10).

### Pi and Anthocyanin Content Assay

Pi content and anthocyanin content were measured by the methods described previously [30].

### Northern and Western Blot Analysis

Total RNA from plants (100 mg, FW) was isolated with TRIZOL® reagent (Invitrogen), and Northern blot analysis was carried out as described previously [28]. In brief, 20 mg total RNA was separated on a 1.2% formaldehyde/agarose gel and blotted on to a nylonmembrane. The membrane was incubated with the α-^32^P-labeled probe at 65°C overnight, and washed under high-stringency conditions. Autoradiography was done at −70°C.

**Figure 8:**
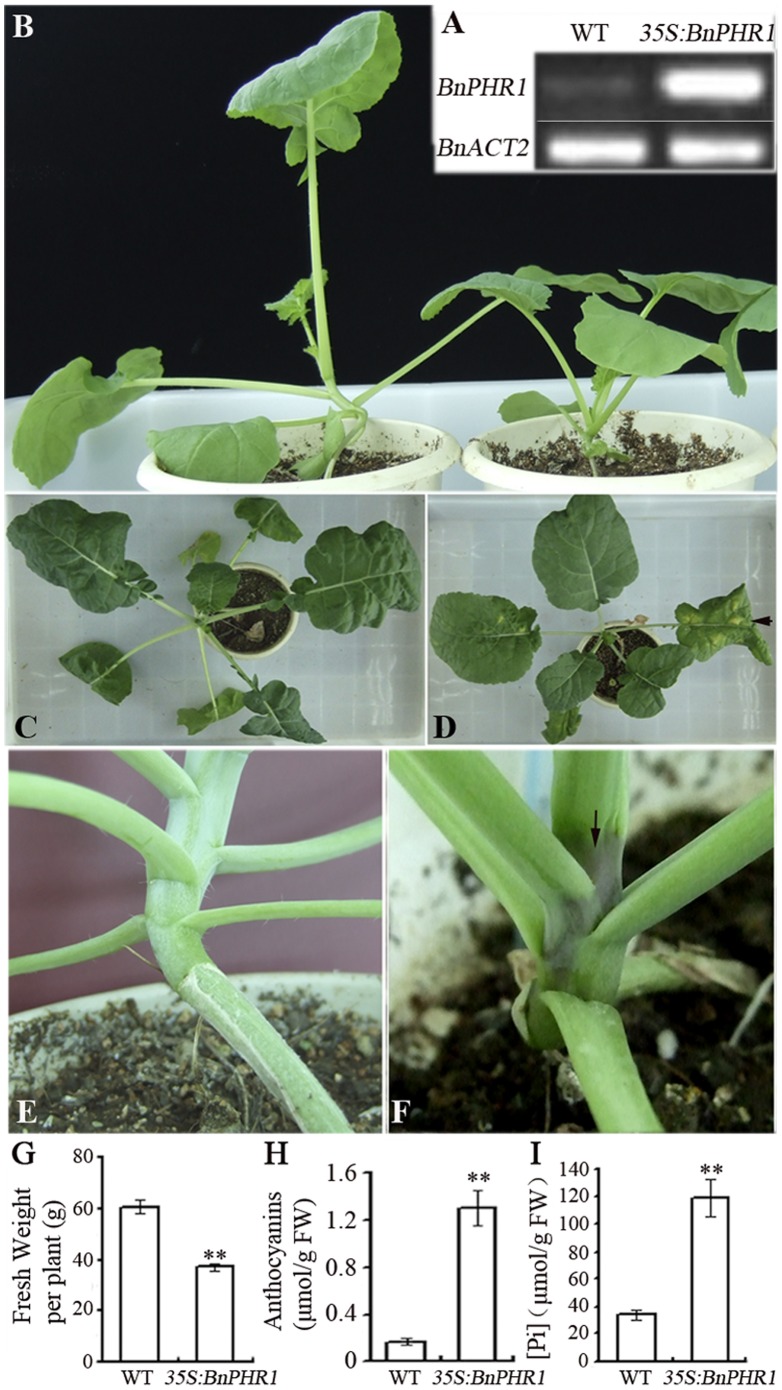
The retarded growth of *BnPHR1* overexpressing *B. napus* in soil. (**A**) The expressing analysis of *BnPHR1* in wild type (WT) and *35S:BnPHR1* transgenic *B. napus* by semi-quantitative RT-PCR. (**B**)**–**(**F**) The growth of wild type and *35S:BnPHR1* transgenic *B. napus* in high Pi conditions. (**B**) The five-week old wild type (left) and *35S:BnPHR1* transgenic (right) *B. napus* grew in soil with high Pi. The seven-week old wild type (**C**) and transgenic (**D**) *B. napus* grew in high Pi conditions in soil. The nodes of basal leaves in wild type (**E**) and *35S:BnPHR1* transgenic (**F**) plants. (**G**) The fresh weight of seven-week-old wild type (WT) and *35S:BnPHR1* transgenic *B. napus* plants in soil. (**H**) Anthocyanins content in shoots of wild type (WT) and *35S:BnPHR1* transgenic *B. napus*. (**I**) Phosphate content in shoots of wild type (WT) and *35S:BnPHR1* transgenic *B. napus*. Error bars indicate standard deviation (*0.01*<p<*0.05, **p<0.01, n = 10).

Total protein was extracted from *35S:GFP* and *35S:BnPHR1-GFP* transgenic Arabidopsis following the protocols described previously [31]. Protein concentration was determined using Bradford reagent (Sigma-Aldrich) with BSA as a standard. For immunoblot analysis, 15 mg of total protein per lane was separated on SDS-PAGE. After electrophoresis, proteins were electrotransferred to Hybond-C membrane (Amersham) using Trans-Blot cell (Bio-Rad), and the anti-GFP antibody (Invitrogen; 1∶2000) was used in protein blot analysis. Primary antibodies were detected using enhanced chemiluminescence peroxidase-labeled anti-mouse secondary antibody (Amersham Pharmacia) and visualized by enhanced chemiluminescence (ECL Advance Western Blotting detection kit; Amersham Pharmacia).

**Figure 9:**
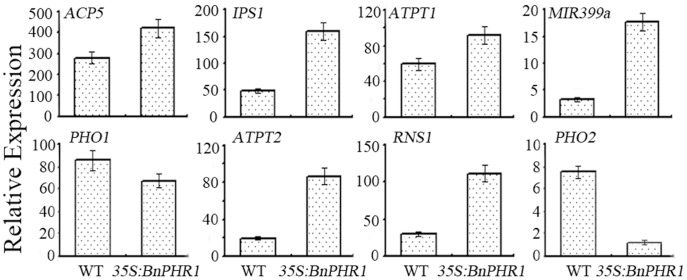
qRT-PCR analysis of Pi starvation response genes in transgenic Arabidopsis. The wild type (WT) and *35S:BnPHR1* transgenic Arabidopsis was cultured in 1 mM Pi, and then the total RNAs isolated from the seedlings were used in real time qRT-PCR analysis. The data are presented as means standard deviation (n = 3).

### Quantitative RT-PCR Analysis

The expression of genes was analyzed by real-time quantitative reverse transcription (RT)-PCR using the fluorescent intercalating dye SYBR Green in a detection system (MJ Research, Opticon 2). The *AtActin2* (*AtACT2*) and *BnActin2* (*BnACT2*) genes were used as standard control in the quantitative RT-PCR reactions. Two-step RT-PCR procedure was performed using a method described earlier [32].

**Figure 10:**
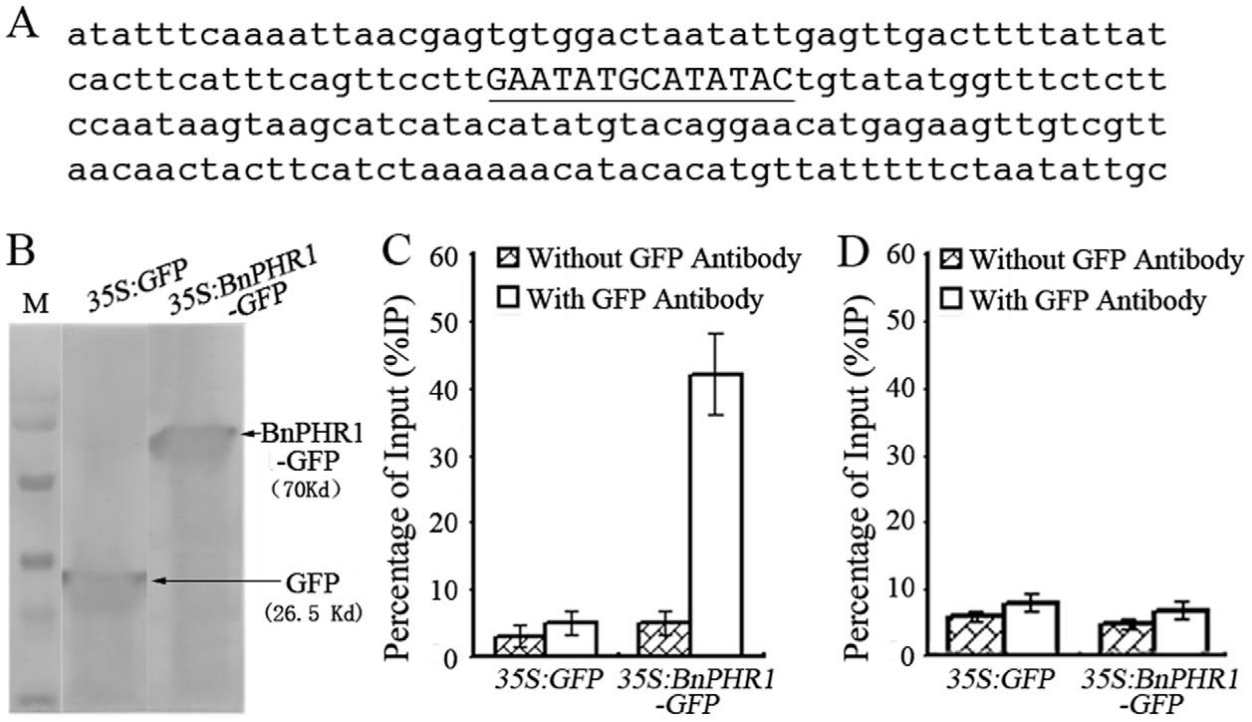
BnPHR1 directly regulating *ATPT2* encoding high-affinity Pi transporter in transgenic Arabidopsis. (**A**) The partial sequence of *ATPT2* promoter containing two overlapped P1BS *cis*-elements (GNATATNC) (uppercase and underlined). (**B**) Western blotting assay of *35:GFP* and *35:BnPHR1-GFP* transgenic Arabidopsis with GFP antibody. (**C–D**) ChIP-qPCR analysis of the *ATPT2* promoter sequence. ChIP assay was performed with chromatin prepared from *35:GFP* and *35:BnPHR1-GFP* transgenic Arabidopsis roots. Genomic DNA fragments that coimmunoprecipitated with GFP antibody were analyzed by real time qPCR using primers amplifying *ATPT2* promoter fragment (**C**) and *ATPT2* coding fragment (**D**) respectively. The experiments were repeated three times, and three replicates were included for each sample in each experiment. The data are presented as means standard deviation (n = 3).

### ChIP Assay

ChIP assay was performed according to the previously described protocol [33]. The seedling roots of *35S:GFP* and *35S:BnPHR1-GFP* transgenic Arabidopsis were cross-linked by formaldehyde and the purified cross-linked nuclei were then sonicated to shear the chromatin into suitably sized fragments. The GFP antibody that specifically recognizes the recombinant BnPHR1-GFP was used to immunoprecipitate DNA/protein complexes from the chromatin preparation. DNA in the precipitated complexes was recovered and analyzed by real time quantitative PCR described above. A 142 bp fragment within the promoter of *ATPT2* was amplified by the specific primer pair AtPT2p1/p2 (Table 1). To ensure the reliability of ChIP data, the input sample and non-antibody control sample were analyzed with each primer set. As the negative control, a 161 bp *ATPT2* genomic DNA fragment (from +1350 to +1510 bp downstream of *ATPT2* start codon) was amplified by a pair of primers ATPT2RT1/3 (Table 1).

**Figure 11:**
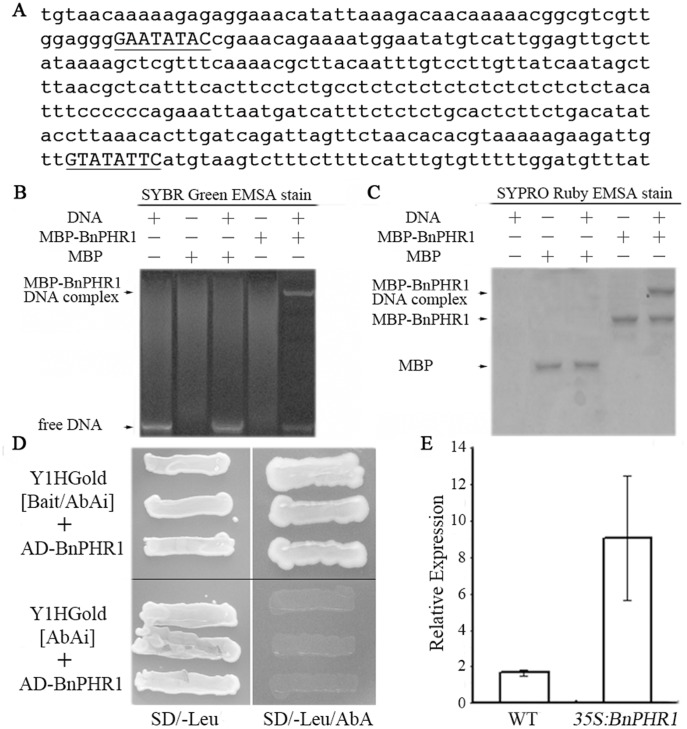
*BnPT2* is the target gene of BnPHR1 in *B. napus*. (**A**) The partial sequence of *BnPT2* promoter. By Genome Walker PCR, a 1 276 bp DNA fragment of upstream region *BnPT2* gene was isolated from *B. napus* genomic DNA. By motif scanning, two P1BS *cis*-elements (GNATATNC) (uppercase and underlined) were found in *BnPT2* promoter sequence. (**B–C**) EMSA of BnPHR1 binding to DNA fragments from the promoter of *BnPT2*. (**B**) The gel was stained with SYBR® Green EMSA stain. (**C**) The same gel as in (**B**) stained with SYPRO® Ruby EMSA stain. The MBP-BnPHR1/DNA complex is observed in both DNA and protein staining. The MBP protein was used as negative control. (**D**) Interaction between BnPHR1 and the *BnPT2* promoter sequence in yeast one-hybrid assays. (**E**) *BnPT2* expression in wild type (WT) and *35S:BnPHR1* trangenic *B. napus* by real time qRT-PCR analysis. Error bars indicate standard deviation (n = 3).

### EMSA Assay

EMSA was carried out using a molecular probes’ fluorescence-based EMSA Kit (Invitrogen). The coding sequence of *BnPHR1* was cloned into pMAL-c2 and inserted downstream from the *malE* gene, which encodes maltose-binding protein (MBP), resulting in the expression of an MBP-BnPHR1 fusion protein. The MBP-BnPHR1 fusion proteins, which were purified from *Escherichia coli* strain BL21 (DE3) using MBP’s affinity for maltose (NEW ENGLAND), was used in protein/DNA binding analysis. A pair of 45-bp oligonucleotides BnPT2p1/p2 (Table 1) from specific promoter of *BnPT2* was synthesized and annealed as DNA probes for EMSA assay. The MBP-BnPHR1 and DNA probes binding reaction was performed in binding buffer (750 mM KCl, 0.5 mM dithiothreitol, 0.5 mM EDTA, 50 mM Tris, pH 7.4) and incubated at room temperature for 20 min. The reaction mixture was separated by non-denaturing polyacrylamide gel electrophoresis. The gel was stained DNA with SYBR® Green EMSA Nucleic Acid Gel Stain and imaged using 254 nm UV epi-illumination. After the staining of DNA, the protein was stained with SYPRO® Ruby EMSA stain.

### Yeast One-hybrid Analysis

To confirm BnPHR1 interacting with *BnPT2* promoter in cells, the Matchmaker Gold yeast one-hybrid system (Biosciences Clontech, Palo Alto, CA, USA) was employed. *BnPT2* promoter (1.2 kb) was inserted upstream of the *AUR1-C* gene in the pAbAi vector, and then efficiently integrated into the genome of the Y1HGold yeast strain by homologous recombination.

The coding sequence of *BnPHR1* was cloned into pGADT7 containing the GAL4 activation domain to create the fusion construct of GAL4-BnPHR1 which was transformed into Y1HGold/BnPT2P reporter yeast strain. Aureobasidin A (AbA) is a cyclic depsipeptide antibiotic, which is toxic to yeast at low concentrations. The resistance to AbA is conferred by *AbAr* gene (*AUR-1C*), which is the reporter on the bait vector pAbAi. When BnPHR1 binds to *BnPT2* promoter sequence, the GAL4 AD activates expression of *AbAr* which allows the cells to grow on media containing the AbA antibiotic.

## Results

### BnPHR1 is a Transcription Activator

From Pi starvation response genes in *B. napus*, one clone (cDNA) was identified as homologous gene of *AtPHR1* and consequently designated as *BnPHR1* (accession number in Gen Bank: JN806156). BnPHR1 composed of 418 amino acids shares 80% identity with AtPHR1. Sequence alignment shows that BnPHR1 is a novel member of MYB-CC gene family in *B. napus* with high conservation in both MYB domain and coiled-coil domain (Figure 1A). *BnPHR1* genomic DNA was 1,847 bp including six introns (Figure 1B).

It also contains a conserved nuclear localization signal (NLS) (Figure 1A). To confirm its nuclear localization, BnPHR1 was fused with an enhanced GFP (eGFP) in transgenic Arabidopsis. As shown in Figure 2A, GFP fluorescence was strongly detected only in the nuclei, demonstrating that BnPHR1 is a nuclear-localized protein.

To analyze the transcription activation ability of BnPHR1, an autonomous gene activation test was performed in yeast system. BnPHR1 was fused to the binding domain (BD) of yeast transcription factor GAL4, and transferred into yeast strain AH109 and Y187 for excluding false positives. On minimal synthetic dextrose (SD) medium lacking Trp, yeast strains with both BD and BD-BnPHR1 vectors grew well. However, on double nutrition-deficient SD medium (SD/−Trp-His or SD/−Trp-Ade), only AH109 strain with BD-BnPHR1 protein could grow well (Figure 2B). The results indicated that BnPHR1 could activate the *HIS3* and *ADE2* reporter genes in yeast strain AH109. The transcription activities of these strains were also measured by α-galactosidase (encoded by reporter gene *MEL1*) activity assay. The results indicated that BnPHR1 could activate *MEL1* reporter gene in yeast strain AH109 and Y187 (Figure 2C).

### Expression Pattern of BnPHR1 in B. napus

*BnPHR1* expression in *B. napus* tissues was analyzed by RT-PCR. The results showed that *BnPHR1* expressed predominantly in roots and flowers (Figure 3A). On the other hand, *BnPHR1* mRNAs are slightly accumulated in shoots and leaves at 72 h after Pi starvation stress (Fig. 3B). The localization of *GUS* expression under the control of *BnPHR1* promoter in transgenic *Arabidopsis* was examined. Histochemical staining of GUS activity revealed that *BnPHR1* promoter was active in roots, especially at the early developmental stage of seedlings (Figure 4A and 4C). In low (1 µM) Pi conditions, GUS activity was still high in roots, but was undetected in tips of elongated roots, especially in tips of primary roots (Figure 4B and 4D). The results suggested that BnPHR1 may play an important role in early root development and its activity in roots is modulated by external Pi.

### The Phenotypic Defects of *phr1* are Rescued by BnPHR1

The *phr1* knockout mutant (SALK_067629) was obtained from the SALK lines collection (Figure 5A). The homozygous *phr1* mutant was identified by PCR (Figure 5B) and then confirmed that *AtPHR1* transcripts were undetectable in mutant lines (Figure 5C). In Pi starvation condition, the distinct phenotype of *phr1* mutant was the impairment of anthocyanins accumulation, which resulted in no dark-purple leaves. The quantitative RT-PCR analysis revealed that *BnPHR1* transcripts were accumulated at high level in *phr1/35S:BnPHR1* plants (Figure 5C). In Pi starvation conditions, the *phr1/35S:BnPHR1* plants displayed dark-purple leaves, like those of wild type, indicating *BnPHR1* shares similar function with *AtPHR1* in Pi starvation and rescues the phenotypic defects of *phr1* mutant (Figure 5D).

### *BnPHR1* Overexpression Affects Growth of Arabidopsis and *B. napus*

*BnPHR1* rescued the phenotypic defects of *phr1* suggested that *BnPHR1* could share similar functions with *AtPHR1* in Pi starvation (Figure 5). To investigate the functions of *BnPHR1* in Pi starvation, *BnPHR1* controlled by 35S promoter was introduced into Arabidopsis. *BnPHR1* transcripts in the transgenic lines were detected by Northern blotting (Figure 6A). On MS medium with high Pi level (1 mM Pi), the growth of *35S:BnPHR1* seedlings was superior to wild type and *phr1* mutant (Figure 6B). On MS medium with low Pi (1 µM Pi), *35S:BnPHR1* seedlings still grew relatively better than that of wild type and *phr1* mutant (Figure 6C). The fresh weight of per *35S:BnPHR1* seedling was more than that of WT and *phr1* in both high Pi and low Pi conditions (Figure 6D). Although there was no significant difference in length of primary roots between wild type and *phr1* mutant in high Pi conditions, the length of primary roots of *35S:BnPHR1* plants was longer than that of wild type and *phr1* mutant in both high Pi and low Pi conditions (Figure 6E). Furthermore, the number of lateral roots of *35S:BnPHR1* plants were superior to those of wild type and *phr1* mutant (Figure 6F). The results indicated that *BnPHR1* overexpressin was in favor of Arabidopsis growth and development in early seedling development. In low Pi (1 µM Pi), *35S:BnPHR1* lines presented dark-purple leaves, especially the dark-purple leafstalks, showing the accumulation of anthocyanins (Figure 6C and 6G).

To investigate effects of *BnPHR1* on Arabidopsis at later developmental stages, the *35S:BnPHR1* plants were grown in soil fertilized with 1.0 mM Pi. The wild type (WT), *phr1* mutant and *35S:BnPHR1* plants were almost identical on development after one week (Figure 7A). However, after two weeks, the growth of *35S:BnPHR1* plants was retarded and the fresh weight of wild type and *phr1* mutants was more than that of *35S:BnPHR1* plants (Figure 7B and 7D). After plants bolting, the size of *35S:BnPHR1* plants was obviously smaller (Figure 7C). These results indicated that the growth of *35S:BnPHR1* plants was accelerated at early stage and then inhibited at later stage of plant development.

To investigate the effects of *BnPHR1* overexpression on *B. napus*, we established a new *Agrobacterium tumefaciens*-mediated *B. napus* transformation system, by which the *35S:BnPHR1* construct was introduced into *B. napus* and over 30 transformants were obtained. The transgenic lines with enhanced *BnPHR1* expression were selected for phenotypic analysis (Figure 8A). The *35S:BnPHR1* and wild type plants were cultured in soil fertilized with 1.0 mM Pi. After three weeks, the *35S:BnPHR1* plants grew much slower than that of wild type (data not shown). After five weeks, the size of the *35S:BnPHR1* plants was much smaller than that of wild type (Figure 8B). After seven weeks, the chlorosis on leaves of *35S:BnPHR1* plants was observed, but not found in wild type (Figure 8C and 8D). After seven weeks, the fresh weight of wild type plants was more than that of *35S:BnPHR1* plants, which was 60.5 and 37.0 g respectively (Figure 8G).

Furthermore, the dark-purple pigment was accumulated nearby the nodes of basal leaves in *35S:BnPHR1* plants, but not in wild type (Figure 8E and 8F). The analysis of anthocyanin content revealed that there was more anthocyanin accumulated in shoots of *35S:BnPHR1* plants than that of wild type plants (Figure 8H).

### *BnPHR1* Overexpression Results in Excessive Phosphate Accumulation in Transgenic Plants

To investigate possible mechanism of the retarded growth of transgenic plants, Pi content in *35S:BnPHR1* Arabidopsis and *B. napus* was measured. The results indicated that Pi content in *35S:BnPHR1* plants was much higher than that of wild type. The content of Pi was 91.4 and 28.3 µmole/g in shoots and roots of *35S:BnPHR1* Arabidopsis, whereas only 23.6 and 5.6 µmole/g Pi in shoots and roots of wild type, respectively (Figure 7E). Similarly, Pi content in shoots of *35S:BnPHR1 B. napus* was about three-fold higher than that of wild type (Figure 8I). The data suggested that overexpression of *BnPHR1* caused excessive Pi accumulation in transgenic plants.

### BnPHR1 Directly Regulates *PT2* Gene Encoding High-affinity Pi Transporter

Expression of eight Pi starvation-related genes (including *IPS1*, one member of the *Mt4/TPSI1* family, *ATPT1* and *ATPT2* encoding a high affinity Pi transporter, *ACP5* encoding an acid phosphatase, *PHO1* involved in Pi loading into xylem, *PHO2* encoding a ubiquitin-conjugating E2 enzyme involved in phosphate starvation response, MIR399a encoding a phosphate starvation-responsive microRNA that targets *PHO2*, and *RNS1* encoding a RNase) in *35S:BnPHR1* Arabidopsis was analyzed by real time qRT-PCR, using gene-specific primers (Table 1). Except *PHO1* and *PHO2*, the expression of the other six genes was increased in *35S:BnPHR1* Arabidopsis (Figure 9). *ATPT2* was significantly up-regulated over four folds in Pi sufficient conditions.

In Arabidopsis, AtPHR1 could bind a P1BS *cis*-element (GNATATNC) in the promoter of its target gene, *AtIPS1* [14]. In *ATPT2* promoter, there was a two-overlapped GNATATNC motif (Figure 10A). To confirm *ATPT2* is one of the target genes of BnPHR1 in *35S:BnPHR1* Arabidopsis, interaction between BnPHR1 and *ATPT2* promoter was detected by ChIP assay. The *35S:GFP* and *35S:BnPHR1-GFP* Arabidopsis was identified by Western blotting assay using GFP antibody (Figure 10B), demonstrating that GFP protein and BnPHR1-GFP fusion protein were constitutively accumulated in transgenic plants and GFP antibody could be employed in ChIP assay. Based on *ATPT2* promoter sequence, a pair of primers ATPT2p1/p2 (Table 1), which could amplify 142 bp DNA fragment including the two-overlapped GNATATNC motif, was designed for PCR in ChIP analysis. On the other hand, a pair of primers ATPT2RT1/3 (Table 1), which could amplify 161 bp *ATPT2* genomic DNA fragment (from +1350 to +1510 bp downstream of *ATPT2* start codon), was designed for PCR in ChIP analysis as negative control. As shown in Figure 10C, BnPHR1-GFP fusion protein strongly interacted with *ATPT2* promoter which encompassed GNATATNC motif, whereas no interaction between GFP and *ATPT2* promoter was detected. And BnPHR1-GFP fusion protein could not interact with the DNA fragment in *ATPT2* coding region (Figure 10D). These results suggested that *ATPT2* is the target gene of BnPHR1 in transgenic Arabidopsis.

We identified some Pi starvation-induced genes (including *BnPT2*) in *B. napus* [30]. The qRT-PCR analysis revealed that *BnPT2* was significantly up-regulated in *35S:BnPHR1 B. napus* (Figure 11E). By Genome Walker PCR, a 1 276 bp fragment of 5′-upstream region of *BnPT2* gene was isolated from *B. napus* genome. *BnPT2* promoter contains two P1BS *cis*-elements (GNATATNC), which are localized from −778 to −771 and −1024 to −1017 bp upstream of *BnPT2* start codon (ATG) respectively (Figure 11A).

To confirm BnPHR1 as a transcription factor binding to GNATATNC elements in *BnPT2* promoter and regulating *BnPT2* expression, protein-DNA binding assay was carried out by EMSA assay. BnPHR1 protein was expressed and purified by pMAL-c2 system. A pair of 45-bp oligonucleotides, BnPT2p1/p2 (Table 1) containing two GNATATNC elements in *BnPT2* promoter were synthesized and annealed to double chain DNA as DNA probes. After stained DNA with SYBR® Green EMSA Nucleic Acid Gel Stain, a large molecular weight DNA band was presented in MBP-BnPHR1/DNA lane, whereas the band was not detected in MBP/DNA, MBP, MBP-BnPHR1 and DNA lanes (Figure 11B). After stained protein with the SYPRO® Ruby EMSA stain, besides a band of MBP-BnPHR1, a larger-size band represented protein-DNA complex was observed in MBP-BnPHR1/DNA lane, but not found in other lanes (Figure 11C). The results suggested that *in vitro* BnPHR1 could bind *BnPT2* promoter.

To validate BnPHR1 binding *BnPT2* promoter *in vivo*, yeast one-hybrid assay was employed. As shown in Figure 11D, yeast cells with the effector (AD-BnPHR1) and *BnPT2* promoter/*AUR1-C* reporter gene grew well on both SD/−Leu medium and SD/−Leu/AbA medium. In contrast, the control cells with AD-BnPHR1 and *AUR1-C* only grew on SD/−Leu medium, but not on SD/−Leu/AbA medium. These data indicated that BnPHR1 could bind *BnPT2* promoter to activate *AUR1-C* gene in yeast cells.

## Discussion

*B. napus* is one of the important oil crops worldwide. Although *B. napus*, as Arabidopsis, belongs to crucifer plant, the conventional genetic transformation of *B. napus* is carried out using various explants, such as stem internodes, stem segments, cotyledonary petioles and hypocotyl segments, which is inefficient and laborious. In this work, based on the floral dip method of *Arabidopsis* transformation [34], an *Agrobacterium tumefaciens*–mediated *B. napus* transformation system was constructed and showed its feasibility to carry on functional genomics in *B. napus*.

Identification and cloning the key regulator of low Pi adaptation is significant for *B. napus* genetic modification. From *B. napus* cDNA library, one *AtPHR1-like* gene, *BnPHR1*, was identified. AtPHR1 as a transcription factor belongs to the members of MYB-CC family with a single repeat MYB domain and a coiled–coil domain [14]. In BnPHR1, the two conserved domains were also present (Figure 1). The high conservation of protein and gene structures between BnPHR1 and AtPHR1 implies both of them are evolved from a mutual ancestor. In BnPHR1, a putative nuclear localization signal was also found in peptides C-terminus (Figure 1). Our data confirmed the nuclear localization of BnPHR1 by GFP fluorescence assay (Figure 2A). An autonomous gene activation test indicated that BnPHR1 has transcription activation ability (Figure 2B), which is consistent with the result of OsPHR1 and OsPHR2, which are homologies of AtPHR1 in rice [17].

It has been reported that neither *AtPHR1* nor *OsPHR1/OsPHR2* are significantly regulated at transcriptional level under Pi starvation [14], [17]. The post-translational modification was hypothesized to be the main activity regulation of AtPHR1, which could be sumoylated by AtSIZ1, a SUMO E3 ligase [19]. Our study revealed that the transcription of *BnPHR1* was slightly induced by Pi starvation in shoots and roots of *B. napus* (Figure 3). The promoter activity analysis in Arabidopsis indicated that *BnPHR1* promoter activity in seedling roots was very strong, implying that the regulation of Pi uptake and homeostasis by BnPHR1 is essential at early stage of plant development. Interestingly, under low Pi condition, *BnPHR1* promoter activity was impaired in tips of both primary roots and the elongated lateral roots. At early stage of plant development, although the decreased promoter activity was found in root tip, the increased promoter activity was also found in other region of root. Generally, the promoter activity was increased in root under Pi deficiency conditions (Figure 4). Previous studies indicated that root is the sensitive organ to external Pi levels, and its architecture was altered in Pi deficiency. Under low Pi conditions, the growth and architecture of plant roots is modified, including promoted growth of lateral roots and inhibited growth of primary roots [3], [4]. The transcriptional pattern of *BnPHR1* in Pi starvation is consistent with the modification of growth and architecture of plant roots to Pi-starvation response. Our results suggested that BnPHR1, an important regulator of Pi starvation response, is modulated in transcriptional level by exogenous Pi. Therefore, regulation of BnPHR1 activity may be involved in both transcriptional level and post-translation modification.

Our data indicated *BnPHR1* could rescue the phenotypic impairment of *phr1* mutant in low Pi condition, indicating that BnPHR1 can substitute AtPHR1 for the signaling of Pi starvation response. In Pi deprivation, a series of genes have been identified as Pi starvation-induced genes in Arabidopsis [7], [15], [35] and some of them were regulated by AtPHR1 [14], [36]. AtPHR1 may be a central positive regulator of most, but not all, Pi starvation-induced genes [37]. The OsPHR1 and OsPHR2, the homologies of AtPHR1 in rice, also could regulate several Pi starvation response genes [17]. In transgenic Arabidopsis, the Pi starvation induced genes (*ACP5*, *IPS1*, *ATPT1*, *ATPT2*, *MIR399a* and *RNS1*) were up-regulated by BnPHR1. *ACP5* encodes an acid phosphatase that promotes phosphate mobilization [38]. *AtIPS1* plays a role in control of P homeostasis by inhibiting the cleavage of PHO2 [39]. *ATPT1* and *ATPT2* encoding Pht1 phosphate transporters play significant roles in Pi acquisition from both low- and high-Pi environments [40]. *MIR399a* encodes a phosphate starvation-responsive microRNA that targets an ubiquitin-conjugating E2 enzyme PHO2 involved in phosphate starvation response [36]. The MIR399/PHO2 pathway as a subcomponent of the Pi-signaling network operates downstream of PHR1 and regulates a subset of Pi-dependent responses in Arabidopsis [36]. The activation of these genes (especially the genes encoding high-affinity Pi transporters) is favor of the uptake and homeostasis of Pi in Arabidopsis. In transgenic *B. napus* with overexpressing *BnPHR1*, the expression of *BnPT2* encoding a high-affinity Pi transporter was significantly increased. Potentially, whether in transgenic Arabidopsis or *B. napus*, BnPHR1 is the positive regulator for the genes encoding high-affinity Pi transporters. Previous study supposed that AtPHR1, as transcription factor, might bind to a conserved *cis* element GNATATNC of *IPS1* promoter [14]. By motif scanning, the *cis* element GNATATNC was found in promoters of *ATPT2* and *BnPT2* genes. By ChIP analysis, we provided the direct evidence that *in vivo* BnPHR1 binds the promoter of *ATPT2* in transgenic Arabidopsis. The EMSA and yeast one-hybrid assay also showed that BnPHR1 binds the promoter of *BnPT2* in *B. napus*. The data confirmed that the genes encoding high affinity phosphate transporters are the targets of BnPHR1 as crucial regulator in phosphate starvation response.

Our data indicated that the content of Pi in shoots of transgenic plants was increased, suggesting that the activation of the high-affinity Pi transporters by BnPHR1 overexpression resulted in the promotion of Pi uptake in transgenic plants. Similarly, previous study revealed that Pi content in shoots of *AtPHR1* overexpression Arabidopsis was increased, whereas Pi content in shoots of *phr1* mutant was decreased in high Pi condition [15]. However, the effects of Pi-level change on growth of the mature Arabidopsis plants are unavailable. In our data, the excessive Pi accumulation in transgenic plants could be the key cause of the retarded growth and smaller plant size, because of Pi toxicity. The chlorosis on leaves of transgenic *B. napus*, one of the characteristics of Pi toxicity, was also observed. These data suggested that BnPHR1 is the important regulator for Pi uptake and homeostasis in plants.

Based on the data presented in this study, we hypothesize that BnPHR1 in the signaling pathway for plant response to low Pi is a crucial component. In Pi starvation, BnPHR1, as a positive regulator, directly activates a subset of genes, including the genes encoding the high-affinity Pi transporters, which promote the uptake of Pi for plant growth.
